# Genetic diversity of *Leishmania amazonensis *strains isolated in northeastern Brazil as revealed by DNA sequencing, PCR-based analyses and molecular karyotyping

**DOI:** 10.1186/1475-9292-6-5

**Published:** 2007-06-21

**Authors:** João Paulo C de Oliveira, Flora Fernandes, Angela K Cruz, Viviane Trombela, Elisângela Monteiro, Anamaria A Camargo, Aldina Barral, Camila I de Oliveira

**Affiliations:** 1 Centro de Pesquisas Gonçalo Moniz, FIOCRUZ, Rua Waldemar Falcão, 121, 40296-710, Salvador, BA, Brazil; 2 Faculdade de Medicina de Ribeirão Preto, USP, Av. Bandeirantes 3900, 14049-900, Ribeirão Preto, SP, Brazil; 3 Ludwig Institute for Cancer Research, R. Prof. Antonio Prudente, 109, 4th Floor, 01509-010, São Paulo, SP, Brazil

## Abstract

**Background:**

*Leishmania (Leishmania) amazonensis *infection in man results in a clinical spectrum of disease manifestations ranging from cutaneous to mucosal or visceral involvement. In the present study, we have investigated the genetic variability of 18 *L. amazonensis *strains isolated in northeastern Brazil from patients with different clinical manifestations of leishmaniasis. Parasite DNA was analyzed by sequencing of the ITS flanking the 5.8 S subunit of the ribosomal RNA genes, by RAPD and SSR-PCR and by PFGE followed by hybridization with gene-specific probes.

**Results:**

ITS sequencing and PCR-based methods revealed genetic heterogeneity among the *L. amazonensis *isolates examined and molecular karyotyping also showed variation in the chromosome size of different isolates. Unrooted genetic trees separated strains into different groups.

**Conclusion:**

These results indicate that *L. amazonensis *strains isolated from leishmaniasis patients from northeastern Brazil are genetically diverse, however, no correlation between genetic polymorphism and phenotype were found.

## Background

*Leishmania *is an intracellular protozoan parasite that infects humans and causes a wide spectrum of diseases known as leishmaniases. Leishmaniases are endemic in 88 countries, where 350 million people live the risk of infection. In the American continent, leishmaniasis is caused by a different number of species and infection with *L. amazonensis*, in particular, produces a wide spectrum of clinical diseases [1]. Severity varies from localized cutaneous leishmaniasis (LCL), the most common clinical manifestation, to diffuse cutaneous leishmaniasis (DCL). LCL, in Brazil, is associated with species belonging to both *Viannia *and *Leishmania *sub-genus whereas DCL is a rare manifestation of leishmaniasis characterized by the presence of nodular, non-ulcerated lesions with abundant parasitized macrophages and the absence of an anti-*Leishmania *cell mediated immune response [2]. In Brazil, DCL is concentrated in the north and northeastern regions, with the highest number of cases being reported in the State of Maranhão [3]. *L. amazonensis *has also been isolated from patients with visceral leishmaniasis (VL) and from patients with muco-cutaneous leishmaniasis (ML), although, in Brazil, these two clinical manifestation are most generally associated with infection by *L. chagasi *and *L. braziliensis *[4], respectively.

A number of methods have been applied to or developed in order to study genetic diversity and relationships within *Leishmania *[5-8]. Among these, we can highlight PCR-based methods such as Random Amplification of Polymorphic DNA (RAPD-PCR) [9] and Simple Sequence Repeat (SSR-PCR) [10]. RAPD-PCR allows the detection of DNA polymorphisms without the need of pre-determined genetic data since it is based on the amplification of genomic DNA under low stringency conditions whereas SSR-PCR is based on amplification of inter-repeat segments using anchored oligonucleotides complementary to the microsatellite repeats. Microsatellites or simple sequence repeats are present in most eukaryotic genomes and consist of tandem repeats of a simple motif of a few nucleotides (< 6) [11]. Both RAPD and SSR-PCR have been used in the study of genetic diversity of *Leishmania *[12-14]. Another PCR-based method to analyze genetic diversity is based on the amplification of the internal transcribed spacers (ITS), located within the rRNA gene array, followed by digestion with restriction enzymes, a method termed "Intergenic Region Typing" [15]. Moreover, the ITS sequences can also be used to generate information useful for phylogenetic reconstruction and molecular evolution studies [16].

Diversity within *Leishmania *has also been investigated at the chromosome level as seen by chromosome size polymorphism [17]. Large-scale chromosomal rearrangements have occurred between Old World and New World species [18] and the chromosome-size polymorphisms are frequently associated with repeated elements present at the telomeres or to the amplification/deletion process of tandemly repeated genes, as in the *L. major *miniexon gene locus, in chromosome 2 [19].

In the present study, we have examined the genetic variability of *L. amazonensis *strains obtained in northeastern Brazil, from patients with different clinical manifestations of the disease, ranging from typical LCL to the rare DCL. In order to do so, different molecular tools such as ITS sequencing, RAPD and SSR-PCR and hybridization of PFGE-resolved chromosomes with gene-specific markers were employed.

## Results

### rRNA sequencing and analysis

**Figure 1:**
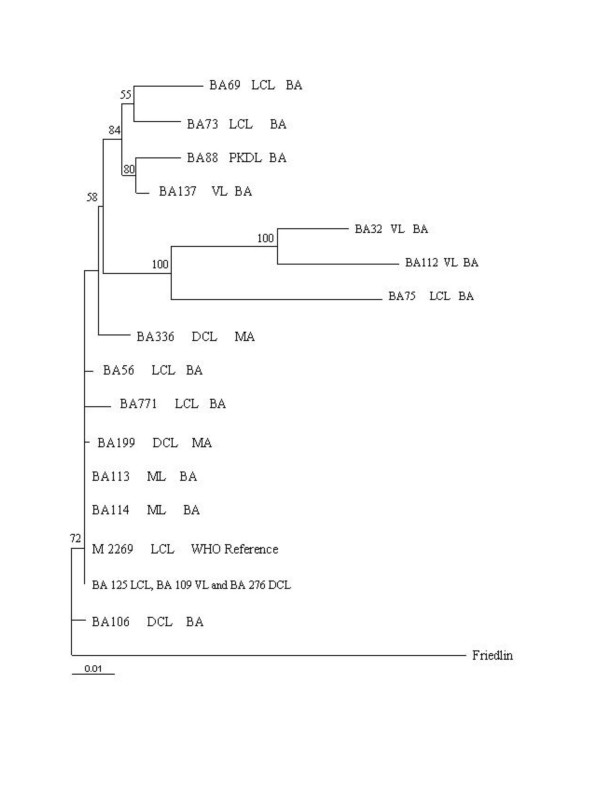
Phylogenetic relationships among *L. amazonensis *strains inferred by Maximum likelihood analysis of the sequences of the ITS1, ITS2 and 5.8 S region. The numbers indicate the percentages with which a given branch is supported in 1000 bootstraps replications. The tree is rooted using *L. major *as outgroup.

To begin examining diversity at the interspecific level, the DNA segment containing ITS 1 and 2, as well as the 5.8 S RNA gene, was amplified by PCR using as template DNA from 17 *L. amazonensis *strains isolated from patients with different clinical manifestations of leishmaniasis, DNA from a reference *L. amazonensis *strain (M2269) and DNA from a *L. major *strain (MHOM/81/IL/Friedlin), which was used as an out-group (Table 1). Except for strain BA115 which was, therefore, omitted from subsequent analyses, PCR amplification yielded a product of ~ 1.2 kb for all strains studied (data not shown). Sequencing of the amplification product revealed 1131 bp orthologous sites and saturation analyses showed no severe saturation, all positions were informative (data not shown). The data set was then subjected to the probability ratio test (LRT) approach. The model that best fit the pattern of nucleotide substitution for our dataset was the HKY85 model [20] plus gamma distribution of α = 0.3641 (indicating heterogeneity of the sites' substitution rates over time). Considering these findings, we performed a phylogenetic signal analysis and found 25.6% of unresolved trees in the likelihood mapping method. As seen in Fig. 1, the tree topology obtained using the HKY model plus gamma distribution revealed two internal nodes, with 100% bootstrap support, in which strains BA32, BA112 and BA75 are clustered. We can only speculate that the clade containing BA32, BA112 and BA75 could be a sister group of the clade containing the BA69, BA73, BA88, and BA137 strains since the boostrap value (58%) was not meaningful.

**Table 1:** Strains of *Leishmania amazonensis *used in this study

**Number**	**International code**	**Origin**^a^	**Pathology**^b^
**1**	MHOM/BR/1985/BA32	BA	VL
**2**	MHOM/BR/1986/BA106	BA	DCL
**3**	MHOM/BR/1987/BA109	BA	VL
**4**	MHOM/BR/1987/BA112	BA	VL
**5**	MHOM/BR/1987/BA113	BA	MCL
**6**	MHOM/BR/1987/BA114	BA	MCL
**7**	MHOM/BR/1987/BA125	BA	LCL
**8**	MHOM/BR/1989/BA199	MA	DCL
**9**	MHOM/BR/1989/BA276	MA	DCL
**10**	MHOM/BR/1973/M2269	PA	LCL
**11**	MHOM/BR/85/BA69	BA	LCL
**12**	MHOM/BR/85/BA73	BA	LCL
**13**	MHOM/BR/85/BA75	BA	LCL
**14**	MHOM/BR/85/BA88	BA	PKDL
**15**	MHOM/BR/87/BA115	BA	LCL
**16**	MHOM/BR/87/BA137	BA	VL
**17**	MHOM/BR/90/BA336	MA	DCL
**17**	MHOM/BR/85/BA56	BA	LCL
**17**	MHOM/BR/00/BA771	BA	LCL

^a^BA, Bahia, MA, Maranhão; PA, Pará.

^b^VL, Visceral Leishmaniasis; LCL, Localized Cutaneous Leishmaniasis; MCL, Muco-Cutaneous Leishmaniasis; DCL, Diffuse Cutaneous Leishmaniasis; PKDL, Post-Kalazar Dermal Leishmaniasis.

All VL isolates (BA137, BA32 and BA112) were clustered in these two clades, with the exception of BA109. The remaining tree branches, however, were found to be unresolved and some strains, such as BA109, BA125 and BA276, were even found to be identical at this locus such. Interestingly, BA106, a DCL case documented outside Maranhão State, where this disease is endemic, remained in a separate node with bootstrap support of 72%. This strain was isolated in Bahia State, in 1986, whereas the other two DCL strains studied (BA199 and BA276) were isolated from patients from Maranhão state, in 1989.

### Genetic polymorphism within *L. amazonensis *strains by RAPD and SSR-PCR

**Figure 2:**
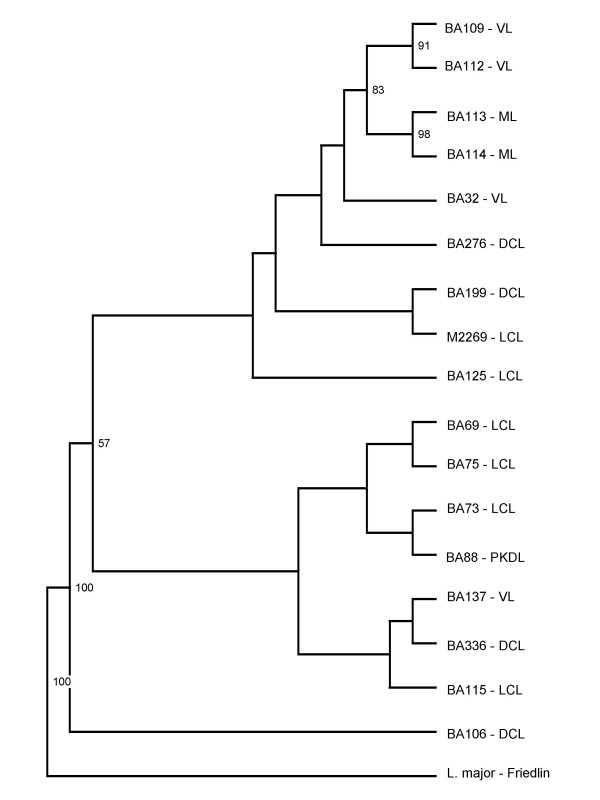
UPGMA dendrogram based on the combined RAPD and SSR-PCR amplification patterns obtained for *L. amazonensis *strains. Bootstrap values above 50% are indicated.

We then compared the topology of phylogenetic tree obtained by ITS sequencing with the dendrograms generated by RAPD and SSR-PCR. Branches observed in the resulting RAPD or SSR-PCR dendrograms were not strongly supported by bootstrap analysis (data not shown). A combined analysis of RAPD and SSR-PCR results is shown in Fig. 2. The grouping of BA109 and BA112 (both obtained from VL patients) as well as the grouping of strains BA113 and BA114 (both from ML patients) were supported by significant bootstrap values (91 and 98%, respectively). In this combined analysis, BA106 (obtained from a DCL strain) also remained in a separate branch, as observed in the individuals RAPD and SSR-PCR dendrograms (data not shown). Overall, either by ITS, RAPD or SSR-PCR, we did not detect any association between genetic polymorphism and geographic origin.

### Chromosome size variations

**Figure 3:**
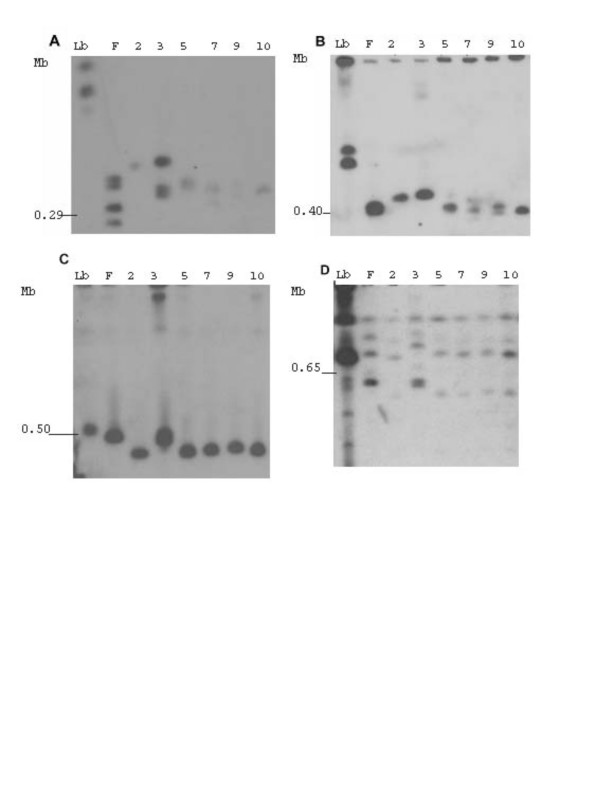
Pulsed Field Gel Electrophoresis resolved *L. amazonensis *chromosomes hybridized with chromosome-specific markers. Following PFGE, gels were blotted and hybridized to radiolabeled probes (A) oligopeptidase; (B) spliced leader RNA gene, (C), DHRF-TS and (D), the genomic fragment from a cosmid clone10E07. Each lane corresponds to one *L. amazonensis *strain, identified by the number reported in Table 1. Lb, *L. braziliensis *; F, *L. major *; Molecular size is shown at the left side of each panel, (Mb). *L. major *chromosome numbers of interest are numbered in the figure.

In order to investigate the degree of chromosome size polymorphism, chrosomomes from *L. amazonensis *BA106, BA109, BA113, BA125, BA276 which are representative of the leishmaniasis clinical manifestation spectrum, *L. amazonensis *M2269, *L. braziliensis *strain (MHOM/BR/94/H3227) and *L. major *Friedlin were separated by PFGE and hybridized to specific chromosome markers. Fig. 3 shows the hybridization patterns observed for each marker used. The hybridization pattern indicates a wide variation in terms of chromosome size for the *L. amazonensis *strains studied. Hybridization with a gene present in both chromosomes 1 and 2 (Fig. 3A) shows variation in terms of chromosome size for the strains tested and, importantly, we observed size polymorphisms between chromosome homologues. A similar result was observed by hybridization with the spliced leader RNA gene present in chromosome 2 (Fig. 3B). In contrast, size polymorphism of chromosome 6, identified by hybridization with the dihydrofolate reductase-thymidulate-synthase (DHFR-TS) gene (Fig. 3C) was less evident among the strains tested. The genomic clone used to identify chromosome 14 (Fig. 3D) has reiterated elements, which are present in other three larger chromosomes of *L. major*, therefore, it allowed the comparison of 4 chromosomes at a time. The results indicate a narrow size variation of these chromosomes within the *L. amazonensis *strains tested.

## Discussion

*Leishmania *infection is characterized by a spectrum of disease manifestations ranging from a relatively benign LCL to the disfiguring ML or the potentially fatal VL and infection with *L. amazonensis *has been associated with these diverse clinical manifestations [1], including DCL. DCL is a rare disease, endemic to Maranhão State, solely associated with *L. amazonensis *infection [21]. In the present work, we examined, for the first time, the genetic diversity of *L. amazonensis *strains from northeastern Brazil, obtained from patients with distinct clinical manifestations of leishmaniasis employing different molecular techniques.

Phylogenetic relationship among strains was inferred based on the sequence comparison of a segment of the rRNA gene locus. The ITS are less conserved between species than rRNA genes and are therefore suitable for the phylogenetic characterization of closely related organisms [22]. The phylogenetic signal analysis showed a number of unresolved trees below 30%, indicating that the data are reliable for phylogenetic inference [23]. Genetic polymorphism within this relatively conserved region has been shown for *L. braziliensis *and *L. naiffi *[15], *L. donovani *[24], *L. aethiopica *[25]. In the present study, ITS sequencing was able to discriminate a subset of the strains; particularly VL strains, although this particular VL clustering was not supported by bootstrap analysis. A conspicuous politomy was observed with the other strains studied, except for strain BA106, indicating a high similarity among the DNA sequences. Similarly, it has been shown that restriction fragment analysis of the ITS region did not reveal differences among *L. mexicana *strains [26].

We then examined the genetic diversity of *L. amazonensis *strains by RAPD and SSR-PCR since both techniques have been used to study genetic polymorphism among *Leishmania *strains [12-14]. In previous reports, genetic polymorphism could be correlated with the geographical origin of parasite strains [12,27-29]or clinical phenotype [30-32]. Recently, we have demonstrated that *L. braziliensis *isolates differing at the genome level also show distinctive infection patterns in BALB/c mice [33]. Herein, no association was found between genetic polymorphism and clinical manifestation of disease or geographical origin of isolate although such the search for these types of correlation was beyond the scope of the present work. On the other hand, we did observe a degree of genetic variability among the *L. amazonensis *strains employed here, particularly BA109, BA112 and BA106. The latter, isolated from a patient with DCL, was consistently grouped separately from all the other strains studied. This was first noted using ITS sequencing and was later confirmed by both RAPD and SSR-PCR. In the phylogenetic and RAPD analysis, BA106 presented the most basal divergence, sharing this position with the *L. major *strain. The occurrence of *Leishmania *stocks phenotipically similar to *L. major*, as assayed by enzyme electrophoresis and monoclonal antibodies, has been documented elsewhere [34]. We could speculate that BA106 is one of these strains, however, in another study, it was typed as *L. amazonensis *by enzyme electrophoresis and monoclonal antibodies[1]. Therefore, further characterization of this strain is needed in order to confirm the identity of BA106.

We also investigated the existence of size polymorphisms in terms of chromosome size since this is widespread phenomenon in trypanosomatids [35]. DNA from *L. amazonensis *strains was separated by PFGE and hybridized with chromosome specific markers, previously anchored to the chromosomes of *L. major *and other Old World species (P. Bastien, A. Cruz et al., unpublished data). We observed size variation of homologous chromosomes of *L. amazonensis*, when compared to *L. major*, as seen with chromosome 2, for example, indicating a possible amplification/deletion of the miniexon array. Biological relevance for the intra-chromosome size variation of the miniexon array has never been reported, although previously observed [19,36,37]. More importantly, chromosome hybridization results for strain BA109 showed a size polymorphism pattern differing from all other strains analyzed, reinforcing the importance of studying the genome plasticity characteristic of *Leishmania*, particularly observed within neotropical strains [38,39]

## Conclusion

In the present work, we have studied the genetic diversity within *L. amazonensis *strains from northeastern Brazil. We show that *L. amazonensis *strains isolated from patients with different clinical manifestations of the disease are genetically diverse and show that this divergence extends to variations in terms of chromosome size. Since *L. amazonensis *is associated with rare or unusual disease presentations such as DCL or VL, respectively, it remains of interest to search for genetic markers that can be associated with these clinical manifestations.

## Methods

### Parasites isolates and genomic DNA preparation

A total of 18 *L. amazonensis *strains were used in this study (Table 1). Strains were obtained between 1985 and 1990 by aspiration of lesions from cutaneous, mucosal, diffuse and post-Kalazar dermal leishmaniasis or by bone marrow aspiration of visceral leishmaniasis patients, each isolate originated from a different human subject [1]. Strains have been maintained in cryopreservation tanks since the time of isolation in culture. For the purpose of this study, isolates were defrosted and cultured until a parasite density of 10^7^/ml was obtained. Species identification was performed by PCR [40] and by ELISA using monoclonal antibodies [41]. For genomic DNA extraction, stocks were grown in 199 medium supplemented with 10% heat inactivated fetal bovine serum (both from Invitrogen), at 26°C. Parasites (10^7^/ml) were harvested by centrifugation and genomic DNA was purified using Wizard^® ^Genomic DNA Purification Kit (Promega). DNA was ressuspedend in 50 μl TE buffer (10 mM Tris, 1 mM EDTA, pH 8.0) and stored at -20°C until used. DNA concentration was estimated by spectrophometry by reading the absorbance at 260 nm. Sample purity was checked by agarose gel electrophoresis and ethidium bromide staining.

### ITS Sequencing and Analysis

The segment containing the ITS 1, 2 and the 5.8 S region, within the rRNA gene locus, were amplified by PCR using primers IR1 (5'-GCTGTAGGTGAACCTGCAGCAGCTGGATCATT-3'), IR2 (5'-GCGGGTAGTCCTGCCAAACACTCAGGTCTG-3') [15] and an internal primer (5'-CGGCGCATGGGAGAAGCT3-3'). PCR products were purified with the CONCERT rapid PCR purification system (Gibco BRL), following the manufacturer's instructions. Amplified PCR products were sequenced in both strands in an ABI377^® ^system using the BigDye Terminator Cycle Sequencing Kit^® ^(Applied Biosystems). Quality analysis and assembling of the sequences were performed using Phred/Phrap/Consed^® ^package [42]. Consensus sequences with Phred quality > 15 were locally aligned with sequences submitted to GenBank using BlastN program [43]. Consensus sequences were submitted to GenBank and are available under the accession numbers DQ300179–DQ300195. Sequence alignment was performed manually using the BIOEDIT software (v5.0.9) [44] and for all sequences, alignment was unambiguous. The mutational saturation was examined using PAUP* (v4.0b10) [45]. The nucleotide substitution model and the gamma distribution were obtained by the likelihood ratio test (LRT) [46], employing the MODELTEST software (v3.0b6) [47]. The phylogenetic signal was tested employing the TREE-PUZZLE (v 5.3) software [48], using the likelihood mapping method [49]. Maximum likelihood (ML) method was then performed in PAUP* (v4.0b10), with a starting tree obtained via Neighbor-Joining algorithm. The reliability of internal branches was assessed using the bootstrap method with 1000 pseudo-replicates. Tree topology was visualized using the TREEVIEW program (v 1.6.2) [50]. The homologous sequence of *L. major *Friedlin strain (MHOM/IL/81/FRIEDLIN) was adopted as the outgroup.

### RAPD and SSR-PCR

RAPD amplification was done as previously described [13], employing one the following primers: 3302 (5'-CTGATGCTAC-3'); 3303 (5'-TCACGATGCA-3') and 3304 (5'-GCACTGTCA-3') (all from Invitrogen). Amplification was performed under the following conditions: an initial denaturation step at 95°C for 5 min, two cycles with annealing at 30°C for 2 min, extension at 72°C for 1 min and denaturation at 95°C for 30 s, followed by 33 cycles in which the annealing temperature was increased to 40°C. The final extension was carried for 5 min at 72°C. SSR-PCR amplification was performed using two primers: [5'-(CA)_8_RY-3'], which amplifies inter-repeat segments [10] and [5'-CAA(CT)_6 _-3'], which amplifies inter-repeat segments as well as the repeat segment [51]. Amplification reactions were performed under the following conditions: 26 cycles of denaturation at 94°C for 30 s, annealing at 50°C for 45 s for the [5'-CAA(CT)_6-_3'] primer or at 57°C for 45 s for the [5'-(CA)_8_RY-3'] primer and extension at 72°C for 7 min. All reactions were performed in triplicate. The amplification patterns were visualized after electrophoresis in a 1.5% ethidium bromide-stained agarose gel.

### Genetic polymorphism analysis

Amplicons were scored by eye and well-defined, reproducible bands (generated in at least three amplification reactions) were used to construct a binary table. Each amplification band was numbered and scored as present (1) or absent (0) and genetic similarity among strains was calculated using Jaccard's similarity coefficient. The relationships between strains were estimated through dendrograms representing RAPD and SSR-PCR data. Dendrograms were constructed based on a matrix of genetic distance through UPGMA [52] using FreeTree (v. 0.9.1.50) [53] and the bootstrap option was used to run 1000 replicates to obtain confidence estimates for the groupings.

### Pulsed Field Gel Electrophoresis (PFGE) and Southern Blot

Chromosomal DNA was prepared in low-melting temperature agarose plugs [54]. Chromosomes were separated by PFGE run on a contour-clamped homogeneous electric field apparatus (CHEF DR II, BioRad). Gels were run at 14°C, at 6.0 V cm^-1 ^for 28 h with a 35.4- to 73.6-s pulse ramp time. Electrophoresed DNAs were transferred to nylon membranes (GeneScreen Plus).

### Probes and hybridization

Four probes were used in this study in order to explore size-polymorphisms of some chromosomes (1, 2, 6 and 14). These were (i) a *L. major *oligopeptidase gene, present in the extremities of chromosomes 1, 2 and 27 of *L. major*; (ii) a *L. major *spliced leader RNA gene, present in chromosome 2; (iii) a *L. major *dihydrofolate reductase-thymidulate-synthase (DHRF-TS) present in chromosome 6 and (iv) a *L. major *genomic fragment from chromosome 14 (cosmid 10E07), which has internal elements repeated in three other larger chromosomes of *L. major *(A.K. Cruz, unpublished results). All probes were labeled with [α-^32^P]dCTP by random priming. Hybridization and washing conditions were performed as described [37] and membranes were exposed at -70°C with an intensifying screen.

## Competing interests

The author(s) declare that they have no competing interests.

## Authors' contributions

JPCO carried out DNA extraction, DNA hybridization, sequence alignment, phylogenetic analysis and dendrogram constructions. FF participated in sequence alignment, phylogenetic analysis and helped to draft the manuscript. AKC participated in the design of the study and helped to draft the manuscript. VT carried out DNA hybridization. AAC participated in the design of the study and helped to draft the manuscript. EM carried out DNA sequencing. AB participated in study design and coordination and helped to draft the manuscript. CIO conceived of the study, participated in its design and coordination and drafted the manuscript. All authors read and approved the final manuscript.
